# TNF-α and IL-10 differentially modulate apoptosis during PRRSV-1 infection of bone marrow-derived dendritic cells

**DOI:** 10.1186/s12917-026-05508-6

**Published:** 2026-05-01

**Authors:** Yanli Li, Enric Mateu

**Affiliations:** 1 Dept. Sanitat i Anatomia Animals, Facultat de Veterinària, Universitat Autònoma de Barcelona, Travessera dels Turons s/n, Cerdanyola del Vallès, 08193 Spain; 2 John Curtin School of Medical Research, The Australian National University, Canberra, ACT, 2601 Australia

**Keywords:** PRRSV-1, Apoptosis, TNF-α, IL-10

## Abstract

Porcine reproductive and respiratory syndrome virus (PRRSV) modulates host immune responses, including apoptotic pathways, during infection. This study examined the role of TNF-α and IL-10 in regulating apoptosis during PRRSV-1 infection of bone marrow-derived dendritic cells (BMDCs), using four isolates with distinct cytokine profiles: 3262 (TNF-α^+^/IL-10^+^), 3249 (TNF-α^+^/IL-10^-^), 2988 (TNF-α^-^/IL-10^+^), and 3267 (TNF-α^-^/IL-10^-^). Early after infection (≤ 24 hpi), infected cells were predominantly non-apoptotic; by 48 hpi, apoptotic bystander cells increased markedly, consistent with a bystander apoptotic process. Neutralizing TNF-α in TNF-α-inducing isolates (3262, 3249) increased the proportion of infected cells and reduced bystander apoptosis, suggesting an antiviral role for TNF-α at the cellular level. In contrast, IL-10 blockade in IL-10-inducing isolates (3262, 2988) increased non-apoptotic infected cells without altering overall infection frequency, suggesting IL-10 modulates apoptotic progression in infected cells. Collectively, these data indicate that TNF-α and IL-10 differentially modulate apoptotic outcomes during PRRSV-1 infection in an isolate-dependent manner.

**Supplementary Information:**

The online version contains supplementary material available at 10.1186/s12917-026-05508-6.

## Introduction

*Porcine reproductive and respiratory syndrome viruses* (PRRSV-1 and PRRSV-2) are enveloped, positive-strand RNA viruses belonging to the genus *Betaarterivirus*, family *Arteriviridae*, within the order *Nidovirales* [3]. They primarily target pig macrophages [4, 8, 9, 28] but can also infect certain in vitro derived CD163^+^ dendritic cells, including bone marrow- (BMDCs) and monocyte-derived (moDCs) dendritic cells [18, 27].

Apoptosis, a tightly regulated form of programmed cell death, interrupts viral replication and helps eliminating infected cells. It operates via two main pathways: the extrinsic pathway, which is activated by death receptors on the cell surface, and the intrinsic pathway, driven by mitochondrial outer membrane permeabilization. The extrinsic pathway primarily activates caspase-8 (and potentially caspase-10), while the intrinsic pathway uses caspase-9. Both pathways eventually trigger the executioner caspase-3 [14]. Some viruses have evolved to inhibit the development of apoptosis during their replication, whereas others induce it at the end of replication to enhance virus release and dissemination [2].

In the case of PRRSV-1, it has been demonstrated that infected macrophages were resistant to staurosporine-induced apoptosis during early infection, suggesting viral interference with apoptotic signaling to enable replication. However, in the later phase, most infected cells underwent caspase-dependent apoptosis, ultimately leading to secondary necrosis [6, 16]. Notably, this late-stage apoptotic response neither facilitated PRRSV assembly nor was essential for viral release from infected cells [6].

While both intrinsic and extrinsic pathways have been implicated, the precise mechanisms underlying PRRSV-induced apoptosis remain incompletely understood. Histopathological analyses of lung and lymphoid tissues indicated that apoptosis predominantly occurred in uninfected cells adjacent to PRRSV-positive macrophages [13, 20, 21, 24, 25] suggesting an indirect mechanism of cell death. One plausible pathway is death receptor-mediated apoptosis [1], in which TNF-α may contribute. Additionally, IL-10 has been implicated in PRRSV-associated apoptosis, although its precise role remains unclear [17, 20].

In this study, we investigated the association of TNF-α and IL-10 with apoptotic outcomes during PRRSV-induced apoptosis and their relationship with PRRSV replication. Four PRRSV-1 field isolates previously shown to induce different TNF-α/IL-10 profiles in BMDCs [12] were chosen: 3262 (TNF-α^+^/IL-10^+^), 3249 (TNF-α^+^/IL-10^-^), 2988 (TNF-α^-^/IL-10^+^), and 3267 (TNF-α^-^/IL-10^-^). These isolates were selected to represent distinct cytokine-inducing phenotypes rather than defined in vivo virulence.

## Materials and methods

### Cells and viruses

Four-week-old piglets were used as donors of alveolar macrophages (AMs) and bone marrow hematopoietic cells (BMHCs). AMs were obtained by bronchoalveolar lung lavage; BMHCs were aseptically isolated from femora and humeri. Cells were tested by PCR to ascertain that they were free of PRRSV, porcine circovirus, and Mycoplasma.

To generate BMDCs, BMHCs were stimulated with 25 ng/ml recombinant porcine granulocyte-monocyte colony stimulating factor (rpGM-CSF) (R&D Systems, Minneapolis, USA) in vitro for 8 days with half of medium changed every three days. The resulting BMDCs were phenotypically characterized by five-color flow cytometry. The majority of cells displayed a CD14⁺MHC-II⁺CD172a⁺CD11R3⁺ phenotype, with heterogeneous expression of CD163, DEC205, and CD1, consistent with previous descriptions of porcine GM-CSF-generated BMDCs [5]. Antibodies and staining details are provided in Supplementary Materials 1 & 2.Four PRRSV-1.1 field isolates were used: 3262 (TNF-α^+^/IL-10^+^), 3249 (TNF-α^+^/IL-10^-^), 2988 (TNF-α^-^/IL-10^+^), and 3267 (TNF-α^-^/IL-10^-^), as previously described in BMDCs [12] (Genbank accession numbers of the four strains are JF276431, JF276433, GQ451686, and JF276435, respectively). Among them, 3262, 3267 and 3249 have been characterized in experimental infections of pigs as moderately virulent, with 3249 and 3267 showing a higher clinical and virological impact [7, 11, 22]. Virulence data are not available for 2988. Viral stocks were the fifth passage in AMs with the same stock used throughout the study. Titration of the produced viruses was performed on AMs. Titers were calculated by the Reed-Muench method [19]. The endpoint of infection was identified by immunofluorescent staining of PRRSV-1 nucleocapsid (N) with a specific antibody (clone 1C5H; Ingenasa, Spain).

### Infection of BMDCs and blockade assay

BMDCs were inoculated with PRRSV-1.1 isolates 3262, 3249, 2988, and 3267 at a multiplicity of infection (MOI) of 0.1 in 15 ml round-bottomed tubes. Mock-inoculated controls received plain RPMI 1640 medium without fetal calf serum (FCS). At 1.5 h post-inoculation (hpi), unbound viruses were washed away with PBS 3⋅, and cells were transferred to 24-well plates at 500,000 cells/well in RPMI 1640 supplemented with 10% FCS. BMDCs inoculated with 3262 (TNF-α^+^/IL-10^+^) were cultured in the presence of excess neutralizing antibodies anti-TNF-α, anti-IL-10 (each at 2 µg/ml; R&D Systems, Spain), or a combination of both. Parallelly, cells inoculated with 3249 (TNF-α^+^/IL-10^-^) and 2988 (TNF-α^-^/IL-10^+^) were treated with anti-TNF-α and anti-IL-10 antibodies, respectively. No neutralizing antibodies were added to cultures inoculated with 3267 (TNF-α^-^/IL-10^-^). For each viral isolate, additional cultures in the presence of recombinant porcine TNF-α (rpTNF-α), rpIL-10 (both at 10 ng/ml; R&D Systems, Spain), or plain medium were also included. All conditions were duplicated and the experiment was independently repeated twice. Cell cultures were harvested at 12, 24, and 48 hpi with cells subjected to flow cytometry staining and supernatants titrated in AMs.

### Assessment of early/late apoptosis

Cells were stained with Alexa Fluor 488 annexin V (1:20; ThermoFisher, Spain) and Near-IR reactive dye (1:1000; ThermoFisher, Spain) in annexin-binding buffer for 15 min at room temperature. After washing, cells were resuspended in cold annexin-binding buffer and kept on ice until analysis. Apoptotic and necrotic populations were defined as follows: Early apoptotic cells (Annexin V⁺/Near-IR^−^), late apoptotic or secondary necrotic cells (Annexin V⁺/Near-IR⁺), and necrotic cells (Annexin V^−^/Near-IR⁺).

### Assessment of cleaved caspase-3 in PRRSV-1-infected BMDCs

Cells were fixed in 4% paraformaldehyde (10 min, room temperature) and permeabilized with ice-cold 90% methanol (30 min, on ice) before blocked with PBS containing 5% FCS and 5% horse serum (20 min, on ice). Subsequently, cells were incubated for 60 min on ice with a polyclonal antibody targeting cleaved Caspase-3 (Asp175) (1:400; Cell Signaling Technology, Spain) and a monoclonal antibody against PRRSV N (1:50; clone 1CH5, Ingenasa, Spain). AF488 conjugated anti-rabbit IgG and AF647 conjugated anti-mouse IgG2b (both were diluted in 1:400; ThermoFisher, Spain) were used as the secondary antibodies (30 min, on ice). Between steps, cells were washed twice with PBS containing 2% FCS. Samples were acquired on a MACSQuant Analyzer 10 (Miltenyi Biotec, Bergisch Gladbach, Germany). The acquired data were analyzed using FCS Express 7 (de novo Software, Glendale, CA, United States). Fluorescence minus one (FMO) and background from secondary antibodies were used for gating.

### Statistical analysis

All figures were made by the GraphPad Prism 9.0 software package (GraphPad Software, La Jolla, CA, United States). Statistical significance was indicated in each figure legend.

## Results

### Infection kinetics

**Fig. 1:**
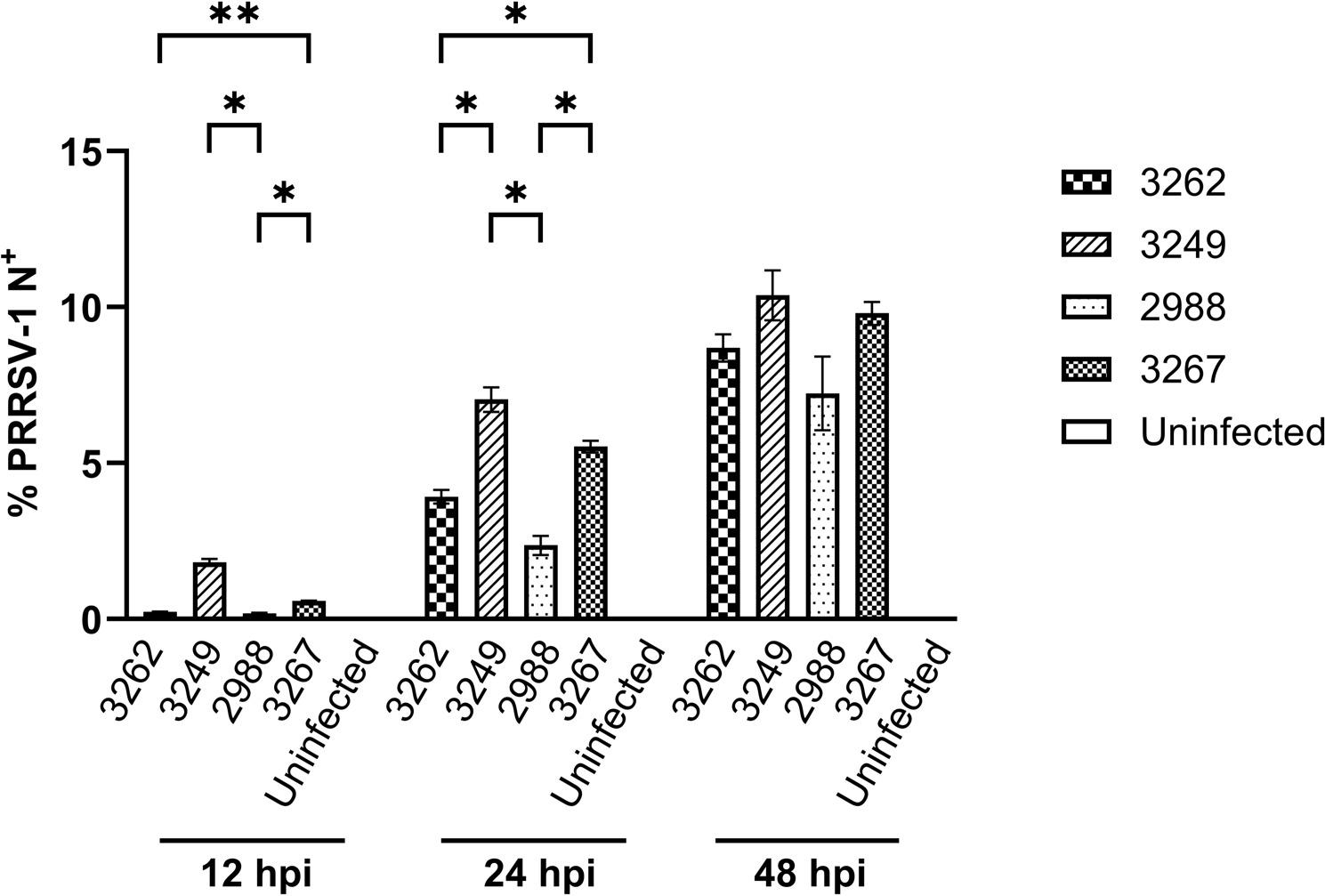
Infection kinetics of four PRRSV-1 isolates in bone marrow-derived dendritic cells (BMDCs). PRRSV-1 isolates 3262, 3249, 2988, and 3267 were used to infect BMDCs at an MOI of 0.1 for 1.5 hours before being washed away. Infection continued in fresh medium until 12, 24, and 48 hours post-infection (hpi) followed by flow cytometry staining of PRRSV-1 nucleocapsid (N). Each isolate was tested in duplicate in two independent experiments. Data are presented as mean ± SD from two independent experiments. Statistical significance was determined using two-way ANOVA with Tukey’s multiple comparisons test (**p < 0.01, *p < 0.05)

The replication dynamics of the four PRRSV-1 strains in BMDCs were assessed at 12, 24, and 48 hpi using an MOI of 0.1. At 12 and 24 hpi, the proportion of infected cells were significantly higher for strains 3249 and 3267 compared to 3262 and 2988 (*p* < 0.05) (Fig. 1); differences disappear at 48 hpi.

### Apoptosis development kinetics

**Fig. 2:**
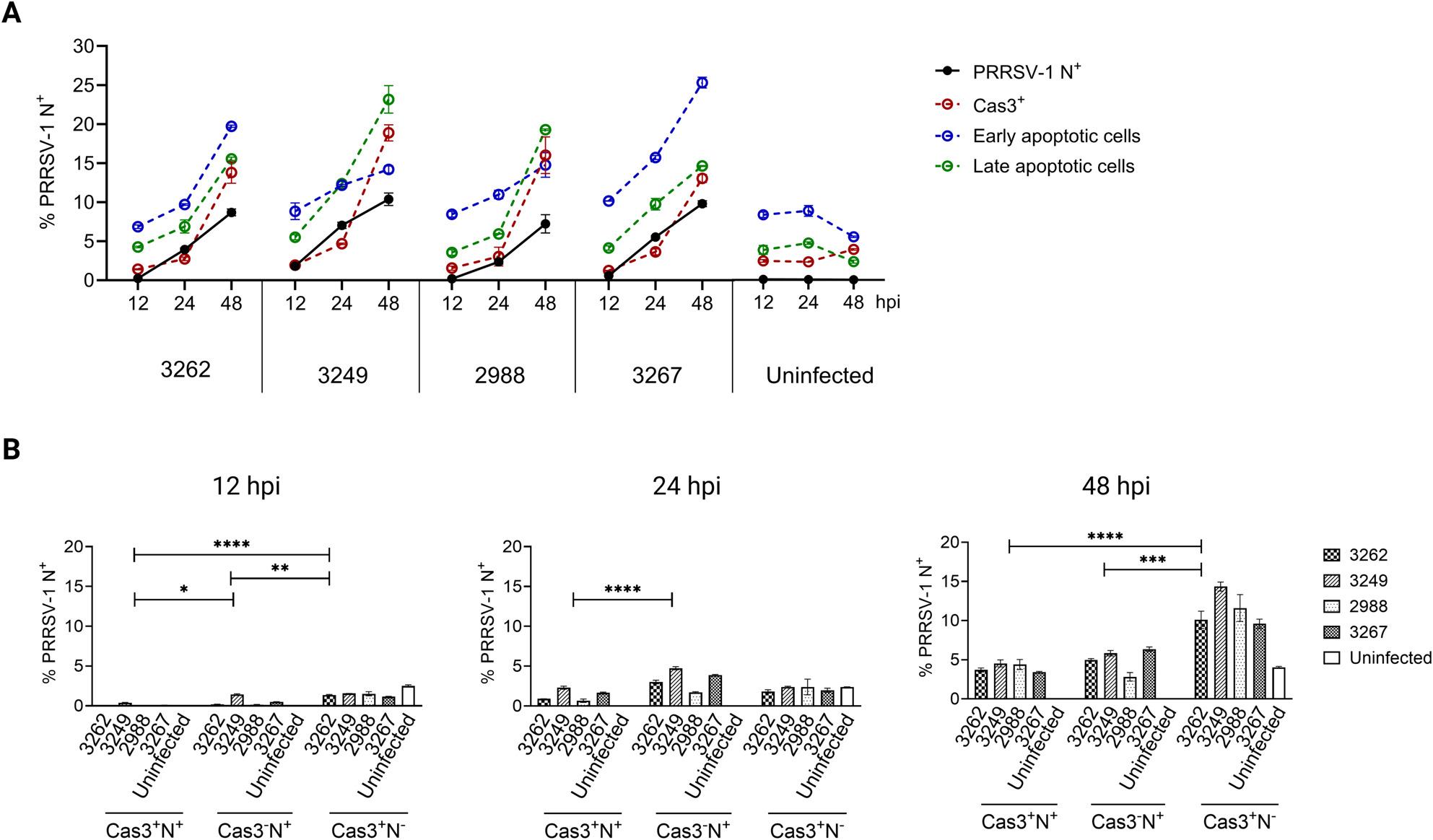
Kinetics of apoptosis in PRRSV-1-infected BMDCs. BMDCs were infected with PRRSV-1 isolates 3262, 3249, 2988, and 3267 at an MOI of 0.1 for 12, 24, and 48 hours with inoculum removed after 1.5 hours. **A** Time-course analysis of infected cells (PRRSV-1 N⁺), caspase-3 activation (Cas3⁺), early apoptosis (Annexin V⁺/Near-IR^-^), and late apoptosis (Annexin V⁺/Near-IR⁺). **B** Proportion of apoptotic infected cells (Cas3⁺N⁺), non-apoptotic infected cells (Cas3-N⁺), and apoptotic bystander cells (Cas3⁺N^-^). Each isolate was tested in duplicate in two independent experiments. Data are presented as mean ± SD from two independent experiments. Statistical comparisons between Cas3⁺N⁺, Cas3^-^N⁺, and Cas3⁺N^-^ groups (mean of the four strains and the uninfected control) were performed using two-way ANOVA followed by Tukey’s multiple comparison test (****p < 0.0001, ***p < 0.001, **p < 0.01, *p < 0.05)

Both early (Annexin V^+^/Near-IR^-^) and late (Annexin V^+^/Near-IR^+^) apoptotic cell population increased over time. But by 48 hpi, apoptosis profiles differed among strains: late apoptosis was more dominant in 3249- and 2988-infected cultures, whereas early apoptosis predominated in 3267- and 3262-infected cultures (Fig. 2A). These differences suggest that apoptosis progression does not directly correlate with infection efficiency.

At 12 hpi, the infected cell population was predominantly non-apoptotic, 0.57% Cas3^-^/N^+^ vs. 0.13% Cas3^+^/N^+^ (averaged across strains) (Fig. 2B). This trend persisted at 24 hpi, where non-apoptotic infected cells (3.34% Cas3^-^/N^+^) outnumbered apoptotic infected cells (1.38% Cas3^+^/N^+^) (Fig. 2B), consistent with an overall higher proportion of infected cells (PRRSV-1 N^+^) than apoptotic cells (Cas3^+^) (Fig. 2A). By 48 hpi, Cas3⁺ cells increased significantly (Fig. 2A), predominantly within the uninfected population (Cas3^+^/N^-^) (Fig. 2B). These results suggest that apoptosis was limited among infected cells early after infection, whereas by 48 hpi bystander apoptosis becomes more prominent.

### TNF-α blockade increased infected-cell frequency while reducing bystander apoptosis

**Fig. 3:**
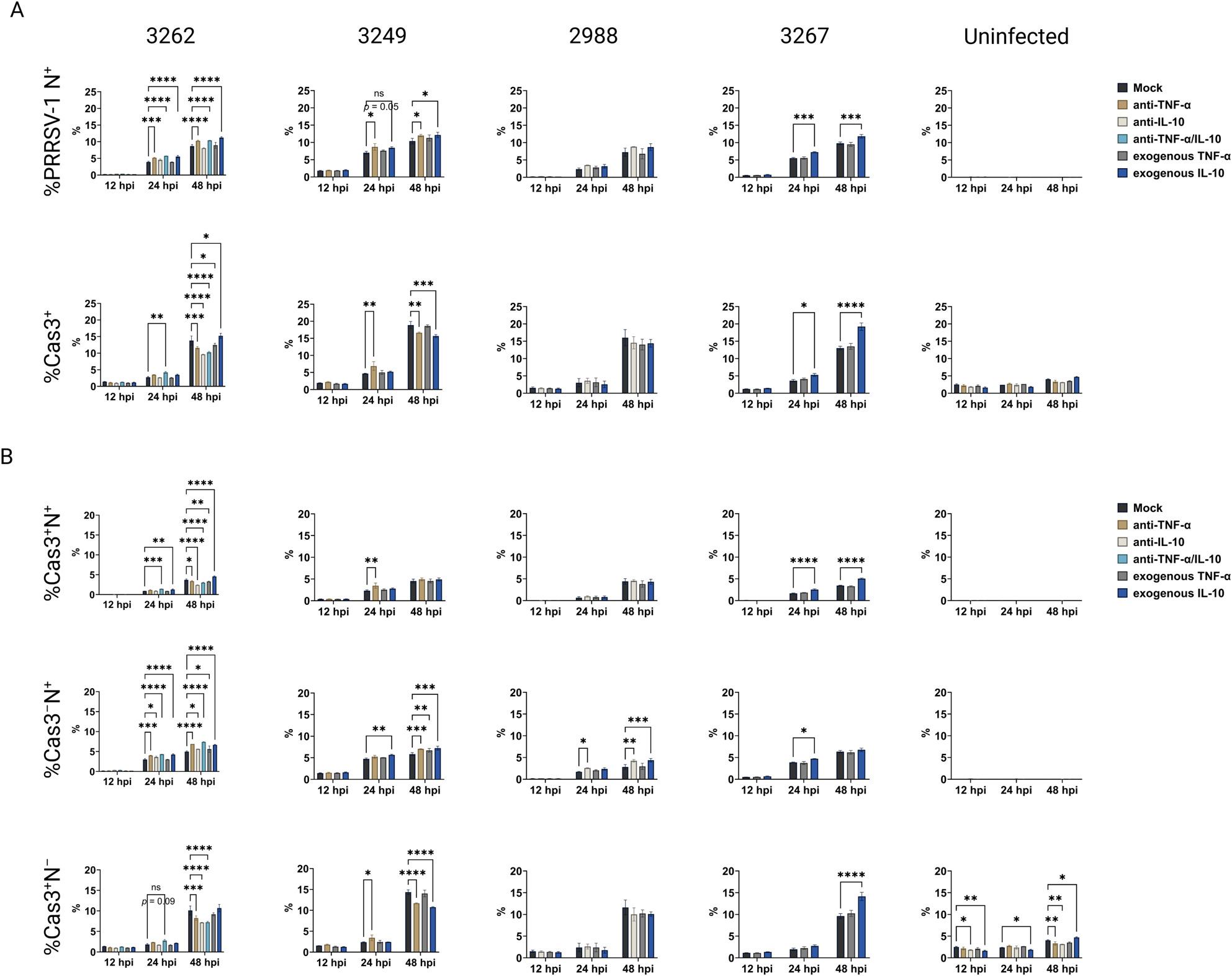
Effect of TNF-α and IL-10 modulation on PRRSV-1 infection and caspase-3 activation in BMDCs. BMDCs were infected in duplicate with PRRSV-1 isolates 3262, 3249, 2988, and 3267 in the presence of 2 µg/ml anti-TNF-α (for 3249 infection), anti-IL-10 (for 2988 infection), or both (for 3262 infection), or 10 ng/ml recombinant porcine TNF-α or IL-10. Cells were collected at 12, 24, and 48 hpi and stained for PRRSV-1 N and cleaved caspase-3. Statistical significance was determined using two-way ANOVA with Dunnett’s multiple comparisons test (****p < 0.0001, ***p < 0.001, **p < 0.01, *p < 0.05). All treatment conditions were performed in duplicate, and the experiment was independently repeated twice. Data are presented as mean ± SD from two independent experiments. **A** Proportions of PRRSV-1-infected (N^+^) and apoptotic (Cas3^+^) cells. **B** Distribution of infected and apoptotic cell subsets: non-apoptotic infected (Cas3^-^N⁺), apoptotic infected (Cas3⁺N⁺), and apoptotic bystander (Cas3⁺N^⁻^)

**Fig. 4:**
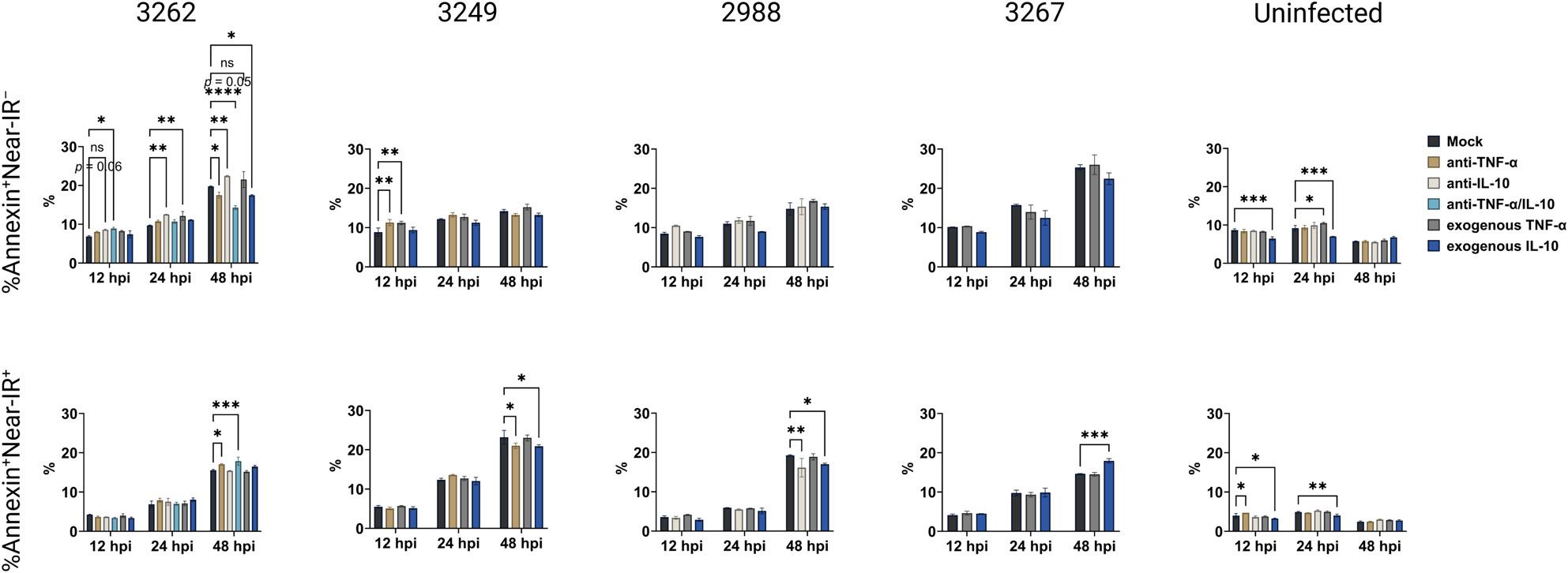
Impact of TNF-α and IL-10 modulation on early and late apoptosis in PRRSV-1-infected BMDCs. BMDCs were infected in duplicate with PRRSV-1 isolates 3262, 3249, 2988, and 3267 in the presence of 2 µg/ml anti-TNF-α (for 3249 infection), anti-IL-10 (for 2988 infection), or both (for 3262 infection), or 10 ng/ml recombinant porcine TNF-α or IL-10. At 12, 24, and 48 hpi, cells were stained with annexin V to detect phosphatidylserine (PS) and Near-IR reactive dye to distinguish live and dead cells. Early apoptosis was defined as Annexin V⁺/Near-IR^-^, and late apoptosis as Annexin V⁺/Near-IR⁺. All treatment conditions were performed in duplicate, and the experiment was independently repeated twice. Data are presented as mean ± SD from two independent experiments. Statistical significance was determined using two-way ANOVA with Dunnett's multiple comparisons test (****p < 0.0001, ***p < 0.001, **p < 0.01, *p < 0.05)

Neutralization of TNF-α during infection with the two TNF-α-inducing isolates, 3262 and 3249, significantly increased the proportion of PRRSV-1 N^+^ cells at both 24 and 48 hpi compared to untreated BMDCs (*p* < 0.05) (Fig. 3A). By 48 hpi, this was accompanied by a reduction in total Cas3^+^ cells for both isolates (Fig. 3A). Subset analysis showed that this reduction was mainly associated with decreased bystander apoptosis (Cas3^+^/N^-^), together with an increase in non-apoptotic infected cells (Cas3^-^/N^+^) (Fig. 3B). This pattern is consistent with reduced bystander apoptosis and preservation of susceptible cells after TNF-α blockade. Despite these changes in infected and apoptotic cell subsets, infectious virus titers in culture supernatants did not differ significantly between groups (Supplementary Fig. 2). Addition of exogenous TNF-α did not reduce the proportion of infected cells but increased early apoptosis (Annexin V⁺/Near-IR^-^) in 3262-infected cultures (Fig. 4), consistent with a pro-apoptotic effect.

### IL-10 blockade increased the frequency of non-apoptotic infected cells, likely by delaying progression to apoptosis

Exogenous IL-10 addition increased the proportion of PRRSV-1 N⁺ cells for all isolates except 2988 (Fig. 3A). In contrast, IL-10 neutralization did not alter the overall proportion of PRRSV-1N⁺ cells in cultures infected with the IL-10-inducing isolate 3262 over 48 h. However, it significantly increased non-apoptotic infected cells (Cas3-/N⁺) at both 24 and 48 hpi, accompanied by decreases in both Cas3⁺/N⁺ and Cas3⁺/N- subsets (Figure 3B). This pattern suggests that IL-10 blockade may delay apoptotic progression in infected cells, namely transition from Cas3^-^/N⁺ to Cas3⁺/N⁺. Supporting this, IL-10 neutralization in 3262-infected cultures consistently increased early apoptotic cells (Annexin V⁺/Near-IR^-^) without a corresponding increase in late apoptotic cells (Annexin V⁺/Near-IR⁺) (Fig. 4), suggesting delayed progression to late-stage apoptosis.

A similar trend was observed for isolate 2988, where an increase in non-apoptotic infected cells (Cas3^-^/N^+^) was noted at both 24 and 48 hpi when IL-10 was neutralized (Fig. 3B). Although the Cas3^+^/N^+^ population did not decline in this isolate (Fig. 3B), a reduction in late apoptotic cells (Annexin V^+^/Near-IR^+^) was seen (Fig. 4).

## Discussion

Using four PRRSV-1 isolates selected for distinct TNF-α/IL-10 induction profiles, our study shows that apoptosis during PRRSV-1 infection of BMDCs is both time dependent and isolate dependent. Early after infection (≤ 24 hpi), infected cells were predominantly non-apoptotic, whereas by 48 hpi apoptosis was enriched in uninfected bystander cells. These findings are consistent with prior reports that PRRSV delayed apoptosis during early replication, possibly allowing efficient replication before cell death [6, 16]. They also fit histopathological observations in PRRSV-infected pigs showing that apoptotic cells are frequently located adjacent to but not co-localized with virus-positive cells [13, 20, 24, 25]. Together, these data support a model in which PRRSV-1 replication initially proceeds in largely non-apoptotic cells, whereas later cell death includes substantial bystanders. Blocking TNF-α during infection with TNF-α-inducing isolates 3262 and 3249 significantly increased the proportion of PRRSV-1 N⁺ cells, consistent with an antiviral role for TNF-α. This increase was accompanied by reduced bystander apoptosis (Cas3⁺/N^−^) and increased non-apoptotic infected cells (Cas3^−^/N⁺), a pattern consistent with TNF-α-associated loss of susceptible, uninfected cells. However, the mechanism remains unresolved. Bystander apoptosis was most evident at 48 hpi, but the present data cannot determine whether the late bystander effect reflects cumulative cytokine exposure, gained expression of death receptors, or other late infection-associated changes. Notably, these changes in infected and apoptotic subsets were not accompanied by corresponding differences in infectious virus titers in culture supernatants, indicating that TNF-α-associated effects were more evident at the level of cellular infection and cell fate than extracellular virus yield. The lack of a clear effect of exogenous TNF-α may reflect saturation of TNF-α-responsive pathways or the induction of anti-apoptotic programs early in infection. In addition, TNF-α can engage caspase-independent death pathways [26], which were not assessed here.

Unlike TNF-α, IL-10 blockade did not change the overall proportion of PRRSV-1 N⁺ cells but shifted cell fates: non-apoptotic infected cells (Cas3⁻/N⁺) increased, whereas Cas3⁺/N⁺ or late apoptotic (Annexin V⁺/Near-IR⁺) cells decreased. This pattern suggests that, during PRRSV-1 infection, IL-10 modulates apoptotic progression in infected cells. Rather than indicating an intrinsically pro-apoptotic role, the effect is more likely linked to IL-10-mediated immune regulation during infection as reported in other infection models [15]. Consistent with this, Flores-Mendoza et al., [10] reported that PRRSV-exposed moDCs showed increased IL-10 production together with apoptosis and reduced MHC-II and CD80/86 expression, indicating impaired antigen-presenting function. Moreover, exogenous IL-10 increased the proportion of infected cells across isolates (except 2988), likely through upregulation of CD163 [23], an essential receptor for PRRSV entry. Together, these findings suggest that IL-10 contributes to PRRSV-1 infection by modulating apoptotic progression and enhancing cellular permissiveness in an isolate-dependent manner. One factor to consider is that blocking or adding a cytokine to the cultures may alter the production of the targeted cytokine, or of other cytokines, through disrupted autocrine or paracrine regulation. This is particularly relevant in homeostatic cytokine networks, where altering one cytokine can trigger compensatory or unintended regulatory responses. Analysis of such cytokine networks is beyond the scope of the present study, which focused on whether PRRSV-induced TNF-α/IL-10 profiles influenced the development of BMDC apoptosis. Notably, the largest apoptotic differences after TNF-α or IL-10 blockade were observed with the dual TNF-α/IL-10-inducing isolate 3262, suggesting that cytokine regulatory networks may be involved and merit further investigation.

Overall, our findings indicate that cytokine signaling contributes to PRRSV-1-associated apoptotic outcomes in an isolate-dependent manner. TNF-α is associated with bystander apoptosis and may limit viral spread, whereas IL-10 modulates apoptotic progression in infected cells. The pronounced strain-specific differences suggest that additional pathways are also involved in regulating PRRSV-induced apoptosis. However, our study assessed apoptosis mainly at the execution stage using cleaved caspase-3. This allowed discrimination between infected and bystander subsets but did not resolve upstream apoptotic signaling (extrinsic versus intrinsic) or alternative regulated cell-death pathways such as necroptosis or pyroptosis. Future studies will be needed to define the downstream TNF-α- and IL-10-associated signaling networks, determine whether multiple cell-death programs are engaged in parallel or sequentially, and assess their broader impact on antiviral immunity in PRRSV-infected pigs.

## Supplementary Information

Supplementary Material 1.

Supplementary Material 2.

## Data Availability

Data produced in this study are available from the corresponding author on reasonable request.

## Abbreviations

PRRSV: Porcine reproductive and respiratory virus
TNF-α: Tumor necrosis factor alpha
IL-10: Interleukin 10
Hpi: Hours post-infection
moDC: Monocyte-derived dendritic cells
BMDC: Bone marrow-derived dendritic cells
AM: Alveolar macrophages
BMHC: Bone marrow haematopoietic cells
MOI: Multiplicity of infection
Cas3: Cleaved caspase 3
N: Nucleoprotein
