# Supplementary material for: TNF-α and IL-10 differentially modulate apoptosis during PRRSV-1 infection of bone marrow-derived dendritic cells
Source: BMC Vet Res. 2026 May 1;22:359. doi: 10.1186/s12917-026-05508-6 (PMC13285190; doi:10.1186/s12917-026-05508-6)
Supplement: Supplementary file 1 — Supplementary Material 1. [file 12917_2026_5508_MOESM1_ESM.docx]

**Supplementary Material 1**:

| **Antibody-fluorophore** | **Clone** | **Isotype** | **Species produced** | **Conjugation** | **Working dilution** | **Supplier** |
| --- | --- | --- | --- | --- | --- | --- |
| Anti-CD14 FITC conjugated | MIL2 | IgG2b | Mouse |  | 1/100 | Bio-Rad |
| Anti-CD172a | BL1H7 | IgG1 | Mouse | LYNX papid RPE antibody conjugation kit (Bio-Rad) | 1/500 |  |
| Anti-SLA II DR | 2E9/13 | IgG2b | Mouse | Zenon mouse IgG2b labeling kits (ThermoFisher) | 1/500 |  |
| Anti-CD11R3 | 2F4/11 | IgG1 | Mouse |  | 1/500 |  |
| Anti-CD1 | 76-7-4 | IgG2a | Mouse |  | 1/50 |  |
| Anti-CD163 | 2A10/11 | IgG1 | Mouse |  | 1/250 |  |
| Anti-DEC-205 (hybridoma supernatant) | 9HZF7 | IgG1 | Mouse |  | 1/1 | Provided by Lab. de Inmunología, CIAD, A.C. |

**The five-color flow cytometry staining of GM-CSF-generated BMDCs.** After viability staining with Zombie Near-IR, cells were stained for CD14, MHC-II, and CD172a, together with CD11R3, CD163, DEC205, or CD1. CD11R3, CD163, and DEC205 were labeled indirectly with a BV421-conjugated secondary antibody. For the combination with CD1, CD172a and CD1 were indirectly labeled with RPE- and BV421-conjugated secondary antibodies, respectively.
