# Supplementary material for: TNF-α and IL-10 differentially modulate apoptosis during PRRSV-1 infection of bone marrow-derived dendritic cells
Source: BMC Vet Res. 2026 May 1;22:359. doi: 10.1186/s12917-026-05508-6 (PMC13285190; doi:10.1186/s12917-026-05508-6)
Supplement: Supplementary file 2 — Supplementary Material 2. [file 12917_2026_5508_MOESM2_ESM.docx]

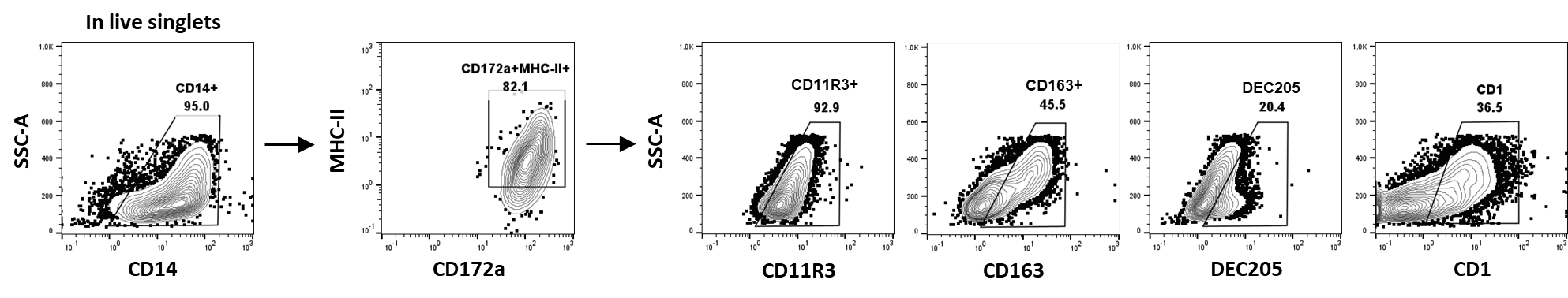


**Supplementary Figure 1. Phenotypic characterization of GM-CSF-generated bone marrow-derived dendritic cells (BMDCs).** Cells were characterized by five-color flow cytometry. Representative contour plots show the gating strategy: live singlets → CD14⁺ → MHC-II⁺CD172a⁺ → CD11R3 (CD11b), CD163, DEC205, or CD1. Most BMDCs displayed a CD14⁺MHC-II⁺CD172a⁺CD11R3⁺ phenotype, with heterogeneous expression of CD163, DEC205, and CD1.

**
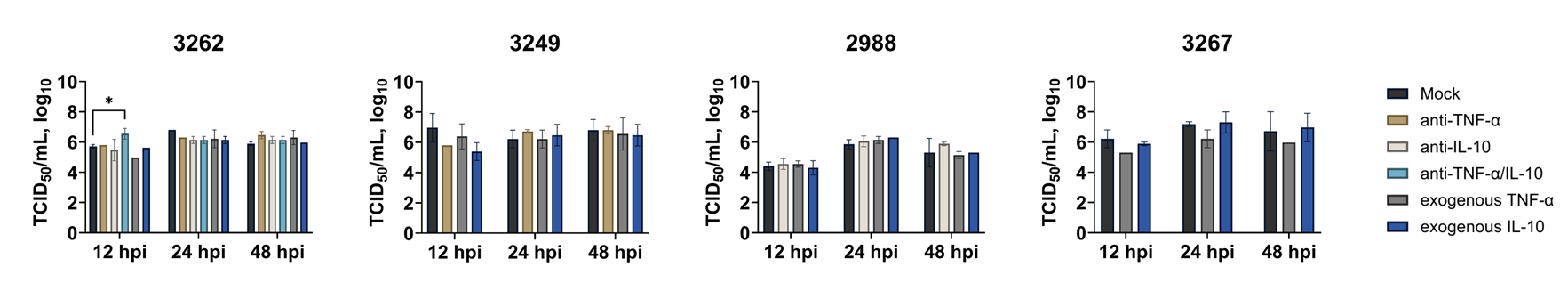
**

**Supplementary Figure 2. Virus titers in PRRSV-1-infected BMDC supernatants under TNF-α and IL-10 modulation.** BMDCs were infected in duplicate with PRRSV-1 isolates 3262, 3249, 2988, and 3267 in the presence of 2 µg/ml anti-TNF-α (3249), anti-IL-10 (2988), or both (3262), or 10 ng/ml recombinant porcine TNF-α or IL-10. Supernatants were collected at 12, 24, and 48 hpi and titrated in alveolar macrophages (AMs). Data are presented as mean ± SD of two independent experiments. Statistical significance was assessed by two-way ANOVA with Dunnett’s multiple comparisons test (**p* < 0.05).
